# Investigation of hot air and foam‐mat dried cress seed gum by FT‐IR, zeta potential, steady shear viscosity, dynamic oscillatory behavior, and other physical properties

**DOI:** 10.1002/fsn3.1514

**Published:** 2020-03-13

**Authors:** Hannaneh Moniri, Reza Farahmandfar, Ali Motamedzadegan

**Affiliations:** ^1^ Department of Food Science and Technology Sari Agricultural Sciences and Natural Resources University Sari Iran

**Keywords:** cress seed gum, drying, foam‐mat, rheology, texture

## Abstract

The effects of different drying methods (hot air drying at 40, 60, and 80°C, and foam‐mat drying) on the characteristics (FT‐IR, zeta potential, conductivity, color, rheology, texture, and emulsifying) of extracted cress seed gum (CSG) have been investigated. The models described the rheological behavior of CSG with high *R* ^2^, but in general Herschel–Bulkley's model has higher values of *R* ^2^ and lower values of RMSE compared to the fitted models. The HD 80 has high amount of viscosity. This means that as the temperature rises, the gel network is getting stronger, and gums from the internal CSG sections have a stronger gel network. Results of strain sweep test demonstrated that storage ($G_{\text{LVE}}^{\prime }$) and loss modulus ($G_{\text{LVE}}^{\prime \prime }$) for all solutions except foam‐mat drying in the linear area showed solid‐like behavior. The parameters of strain sweep (G_f_, τ_f_, τ_y_, $G_{\text{LVE}}^{\prime \prime }$, $G_{\text{LVE}}^{\prime }$, Y_LVE_) increased with increasing temperatures. Frequency sweep test showed that storage ($G_{\text{LVE}}^{\prime }$) was greater than loss modulus ($G_{\text{LVE}}^{\prime \prime }$) and samples have a solid behavior but foam‐mat drying exhibited liquid behavior. Increasing temperature has a direct impact on texture, so hardness and adhesion are increased consequently. Generally, CSG has good emulsifying and foaming characteristics, but no significant difference was observed.

## Graphical abstract

Different drying methods of cress seed gum could produce powders with different functional properties which prepare beneficial information regarding their application in food and pharmaceutical industries.

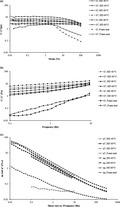

## 1 INTRODUCTION

Hydrocolloids or gums are hydrophilic polymers with a high molecular weight and long chain that are dispersive, completely or partially dissolved in water. They swell in water, and colloid systems with different structures are produced (Williams & Phillips, 2009). Food hydrocolloids are used as thickening agents, gelling, water holding, dispersing, stabilizing, film forming, foaming, and as tissue modifiers in foods also. They are usually used in low concentrations and affect the tissue, rheology, and sensory properties of the final product. Hydrocolloids increase the stability of emulsion by increasing the continuous phase viscosity or interaction with surface‐active substances. One of the most sources of plant hydrocolloids is mucilaginous substances. Mucilage is a hydrophilic polysaccharide and heterogeneous branched which can be thick and sticky when dissolved in water. Mucilage is produced in various parts of the plant, such as leaves, seeds, and buds (Razavi, 2019). *Lepidium sativum* L. or garden cress (GC) is a member of the Brassicaceae family which extensively grows in the Middle East, Europe, and United States (Karazhiyan et al., 2009). When water enters the polyuronide chain, the chain is hydrated and swollen, resulting in dispersion of cellulose micelles. By swelling the CSG in the water, a large number of mucilaginous components are formed in the water and a clear gel is formed around the gum. The amount of oil (solvent extracted) in *Lepidium sativum* seeds is 21.54%. α‐Linolenic (34%) and oleic acids (22%) are high in garden cress seed oil (GCO). Important phytochemicals in cress seed are alkaloids, flavonoids, cardiotonic glycosides, glucosinolates, sterols, tannins, and triterpene which create pharmacological properties in it. Drying and its impact on functional properties have been known for many years. Drying is a complex process that transmits heat and mass along with physicochemical changes (Jafari, Azizi, Mirzaei, & Dehnad, 2016). Functional and nutritional compounds like antioxidant and phenolic composition are increased by drying. Creating a balance between drying time and product quality is essential to maintain its quality and functional characteristics. Most drying methods are performed in two separate steps. In the first stage, the moisture is removed at a constant rate and transported to the surface at the same rate as evaporation occurs. The next stage is falling of speed, which occurs at various rates as a product phase changes (Malekjani, Jafari, Rahmati, Zadeh, & Mirzaee, 2013). The effect of drying on the properties of foods includes loss of color, loss of aroma, tissue changes, nutritional value, and physical appearance. Although high temperature reduces the drying time, it causes heat damage to surface and higher energy consumption. Foam‐mat drying has a high speed due to the open structure of the foam, and a large expose surface to drying, so more moisture is removed. The factors that affect the drying rate are air bubble collaboration which will damage the process of drying if collapsed. The composition of food, type, and concentration of foaming agent affect characteristics of the foam and viscosity, while low surface tension affects the stability of the foam (Krasaekoopt & Bhatia, 2012). The application of foam‐mat drying is present in heat‐sensitive foods (viscous, sticky, and high sugar content) which are not to be dried in other ways, such as spray dryer. Foam‐mat drying has been considered for simplicity, speed, and efficiency. In this study, the effect of foam‐mat drying method and hot air drying method (40, 60, and 80℃) on the rheology, emulsion, and textural properties of CSG has been studied.

## 2 MATERIALS AND METHODS

### 2.1 Cress seed gum extraction

Cress seeds were purchased from a local market in Babol, Iran. CSG powder was obtained according to Karazhiyan, Razavi, and Phillips (2011). The cress seeds were cleaned and soaked in distilled water with pH 7, ratio of 30:1, 35℃ for 15 min. The mucilage of cress seeds was separated by passing seeds through the extractor (Pars Khazar 700P, Rasht, Iran), which scratched the surface of gum layer. The mucilage of the extracted cress seed was then filtered and collected. After this, the collected mucilage was dried with different drying methods.

### 2.2 Drying process

#### 2.2.1 Hot air drying

The cress seed mucilage was dried by hot air oven (convection oven, Memmert, model ULM400, Germany) at three temperatures (40, 60, and 80°C) for 24 hr.

#### 2.2.2 Foam‐mat drying

This method was carried out according to Djaeni, Prasetyaningrum, Sasongko, Widayat, and Hii (2015) with some modifications. Initially, 2% albumin powder was used as a foaming agent and 0.1% methyl cellulose as a stabilizer; 9.5 g extracted CSG and 40 ml water were mixed (Black & Decker, M220) for 5 min at room temperature. The produced foam was quickly poured into a cylinder. Afterward, it was poured into a container with a thickness of 0.5 cm and then dried in oven (convection oven, Memmert, model ULM400) at 80°C for 2 hr.

#### 2.2.3 FT‐IR spectroscopy

The FT‐IR analysis (Desktop FTIR Spectrometer, CARY 630 Model, Agilent) was used to determine the functional groups in CSG powders. The FT‐IR measurement was in a range from 4,000 to 650 cm^−1^. According to Karazhiyan et al. (2011) with a few changes, the solutions were made up to 0.5% (w/w) and placed on the stirrer for 30 min to become homogeneous. Then, three volumes of 96% ethyl alcohol were added to one volume of CSG solutions and placed at room temperature for 2 hr. The precipitate was dried in oven (convection oven, Memmert, model ULM400) at 50°C overnight. Then, the samples were pulverized using a mill (Bosch, MKM6000) and passed through a mesh 18 to obtain uniformity of particles. The dried samples were kept in a desiccator until the analysis was done.

#### 2.2.4 Zeta potential and conductivity

Zeta potential was performed using a device (Zetasizer, Malvern Instrument) at 25°C and pH 7. For this purpose, the CSG solution (0.1%, w/v) was stirred at room temperature for 24 hr. The conductivity was also measured at the same time. Samples were measured at least twice.

#### 2.2.5 Color measurement

The color measurement of gum powders was performed using a device equipped with digital camera. The samples were placed in the middle of the device, and images were converted using the color‐space‐conversion program from color space RGB to LAB. L* is the lighting index, which ranges from zero to 100. The parameter a* is positive for red colors and negative for green colors, while the parameter b* is positive for yellow colors and negative values for the blue ones (Granato & Masson, 2010). Chroma (C*) is defined as a quantitative attribute. The higher value of the chroma indicates the higher intensity of color that human perceives. Chroma was calculated using following equation:

(1) $$C^{*}=\sqrt{a{*}^{2}+b{*}^{2}}$$

Hue angle is a qualitative attribute which is defined colors based on redness and greenness. An angle of 0° or 360° represents red hue, but angles of 90°, 180°, and 270° represent yellow, green and blue hues, respectively. The hue angle of powders was calculated based on the following procedure (Pathare, Opara, & Al‐Said, 2013):

(2) $$h^{*}=\tan^{-1}\left(\frac{b*}{a*}\right)\;\;\;\;\;\;\;\;\;\;\;\;\;\;a^{*}>0,b^{*}>0$$

(3) $$h^{*}=180^{\circ }+\tan^{-1}\left(\frac{b*}{a*}\right)\;\;\;\;\;\;\;\;\;\;\;a^{*}<0$$

(4) $$h^{*}=360^{\circ }+\tan^{-1}\left(\frac{b*}{a*}\right)\;\;\;\;\;\;\;\;\;\;\;\;\;a^{*}>0,b^{*}<0$$

Total color difference (ΔE*) shows the color difference between stored and control samples. (ΔE*) divided into several intervals such as very distinct (ΔE > 3), distinct (1.5 < ΔE < 3), and small difference (1.5 < ΔE) (Pathare et al., 2013).

(5) $$\Delta E^{*}=\sqrt{\Delta a{*}^{2}+\Delta b{*}^{2}+\Delta L{*}^{2}}$$

The browning index was calculated by the following procedure:

(6) $$BI=100\times \left(\frac{X-0.31L}{0.17}\right)$$

(7) $$X=\frac{\left(a*+1.75L\right)}{\left(5.645L+a*-3.012b*\right)}$$

### 2.3 Rheological measurements

A physica‐MCR 301 rheometer (GmbH, Anton Paar, Austria) was used to study the rheological properties of CSG. The rheometer was equipped with a serrated parallel plate system (c P50‐2‐SN 39,797; d = 0.206 mm). The samples were rested for 10 min at 20℃. For rheological measurement, the solution of CSG was prepared with concentration (1.5%) and 0.02% sodium azide (NaN_3_) which was added to the solution as microbial additive. To complete hydration, the solutions were placed at 4°C overnight.

#### 2.3.1 Shear rate dependency

Different flow models such as power law, Herschel–Bulkley, Casson, and Bingham were used to describe the steady shear rheological properties of CSG solutions at a shear rate of 0.01–300/s (20℃).

(8) $$\text{Power law}:\tau =K_{\rho }\;{\dot{\gamma }}^{n_{p}}$$

where *K*_p_ is the consistency coefficient (Pa s^n^), n_P_ is the flow behavior index (dimensionless), τ is shear stress (Pa), and $\dot{\gamma }$ is shear rate (s^− 1^).

(9) $$\text{Herschel}-{\text{Bulkley}}^{\prime }s:\tau =K_{H}{\left(\dot{\gamma }\right)}^{n_{H}}+\tau_{0H}$$

where *K*_H_ is the consistency coefficient (Pa s^n^) and n_H_ is the flow behavior index (dimensionless). K_H_ indicates the magnitude of viscosity. τ_0H_ is the yield stress (Pa) of Herschel–Bulkley model.

(10) $$\text{Casson model}:\tau^{0.5}=K_{0c}^{0.5}+k_{c}{\left(\dot{\gamma }\right)}^{0.5}$$

K^2^_0c_ is Casson yield stress (τ_0_ *_c_*, Pa) and k_c_^2^ is Casson plastic viscosity (*η_c_*, pa s).

(11) $$\text{Bingham}:\tau =\eta_{\beta }\dot{\gamma }+\tau_{0\beta }$$

*η_β_* (Pa s) is called the viscosity of Bingham, and τ_0B_ (*pa*) is yield stress of Bingham.

#### 2.3.2 Small dynamic oscillatory measurements

##### Strain sweep

The parameters determined by strain sweep tests (0.01%–100%, 1 HZ, 20 ℃) are the storage modulus ($G_{\text{LVE}}^{\prime }$), viscous modulus ($G_{\text{LVE}}^{\prime \prime }$), loss tangent (tan_δ_), τ_y_ (yield stress), τ_f_ (flow‐point stress where two modulus become equal G′ = G″), and limiting value of strain (Y_LVE_) at the LVE region (Yousefi & Razavi, 2015).

##### frequency sweep

The measurement of frequency tests in a constant strain (0.3% pa) was in the range of 0.1 to 10 Hz at 20°C. The parameters that are measured through this test are the viscous modulus (G″), the storage modulus (G′), the loss tangent (Tan(δ)), the complex viscosity (ɳ*), and the slope of complex viscosity.

### 2.4 Texture measurements

To perform texture profile analysis (TPA) test with texture analyzer (CT3 10k, Brookfield, USA), first the solutions of 7% CSG were prepared and placed at room temperature to complete hydration for 48 hr. To prepare the gels, a syringe (20 mm inner diameter; 8.5 cm length) was used and the gel was poured into it. The TPA test was performed using a cylindrical probe with 35 mm diameter at 30 mm/min at 50% of its original height, and the gel was removed from the syringes with 20 mm length. The parameters of texture such as hardness and adhesiveness were measured.

### 2.5 Emulsifying properties

The method of Sciarini, Maldonado, Ribotta, Perez, and Leon (2009) is used to obtain the emulsion capacity and emulsion stability. Sunflower oil (6ml) was used to create oil in water emulsions and was gradually added to CSG solutions (1% (w/w), 60 ml) and then blended on roller mixer at 559 *g* for 10 min. Then, it was homogenized (Ultra‐Turrax, D500, DRAGON LAB) at 12879 *g* for 1 min. Finally, the suspensions were centrifuged at 2236 *g* for 10 min (T450GG urum tajhiz gostar). The emulsifying capacity was calculated as:

(12) $$\text{Emulsion capacity}\;\left(\%\right)=\left(\frac{e_{v}}{t_{v}}\times 100\right)$$

where e_v_ is emulsion volume and t_v_ is total volume.

The emulsion was placed in water at a temperature of 80°C for 30 min in order to determine its stability and then centrifuged at 2236 *g* for 10 min. Emulsion stability was calculated as:

(13) $$\text{Emulsion stability}\;\left(\%\right)=\left(\frac{f_{\mathrm{ev}}}{i_{\mathrm{ev}}}\times 100\right)$$

where f_ev_ is the final emulsion volume and i_ev_ is the initial emulsion volume.

### 2.6 Foaming properties

To prepare the foam, 2% ovalbumin was added to gum solution (0.1%, 45 ml) and whipped strongly for 4 min. Then, foam capacity and foam stability  were calculated according to the following equations:

(14) $$\text{Foam capacity}\;\left(\%\right)=\left(\frac{\text{volume after whipping}-\text{volume before whipping}}{\text{Volume after whipping}}\right)\times 100$$

(15) $$\text{Foam stability}\;\left(\%\right)=\left(\frac{(\text{Foam volume after 30 min})\text{ml}}{(\text{Initial foam volume})\text{ml}}\right)\times 100$$

### 2.7 Statistical analysis

Statistical analysis of data was performed using SAS 9.3.1 software. The data were analyzed by Duncan's multiple range test (*p* level of < 0.05). MATLAB (2014b), and Physica Rheometer Data Analysis software (Rheoplus/32, version V3.40) was used to evaluate rheological parameters.

## 3 RESULT AND DISCUSSION

### 3.1 FT‐IR spectroscopy

**Figure 1:**
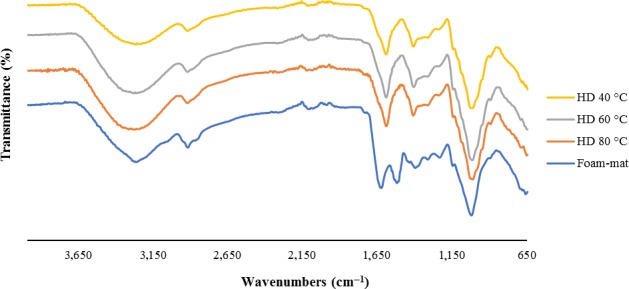
Fourier transform infrared spectroscopy (FT‐IR) of drying methods. HD (hot air drying)

The result of FT‐IR related to CSG is illustrated in Figure 1. All normal bands and peaks that related to polysaccharides properties are shown in the FT‐IR spectrum. Carbohydrates in CSG have the finger print area in the wave numbers between 800 and 1,200 cm^‐1^, and it can be used to determine differences in the structure of samples (Nep & Conway, 2011). Peaks in this area represent little differences between the samples and no change in structural characteristics. The vibration of O‐H happened in the range of 3,200 cm^−1^ between 3,400 and 3,000 cm^−1^ which indicates some features such as free hydroxyl groups stretching bonds that happened in vapor phase. The C‐H vibration is in the range of 2,800–3,000 cm^−1^ wavenumber, which includes CH, CH_2,_ and CH_3_ stretching and in the form of symmetric and asymmetric bending vibrations that sometimes overlap with O‐H (Razavi, Cui, Guo, & Ding, 2014). Existence of Amid Ι (stretching vibrations of C = O and C‐N groups) and Amid Ⅱ (mainly from N‐H bending) in absorbent wavelengths between 1,700–1,600 cm^−1^ and 1,600–1,500 cm^‐1^ in CSG proves the presence of protein (Naji‐Tabasi, Razavi, Mohebbi, & Malaekeh‐Nikouei, 2016). The C–O–H bonds and C–O, C–O–C glycosidic exist in the area between 800 and 1,150 cm^−1^ (Razavi et al., 2014). The asymmetrical vibration is attributed to the peak of 1,600 cm^−1^, and the symmetrical COO− stretching vibrations are attributed to the peak of 1,400 cm^−1^ (Jindal et al., 2013).

### 3.2 zeta potential and conductivity

Zeta potential is used to Determine the amount and type of electrostatic reactions that occur simultaneously in biopolymers. In Table 1, CSG solutions with a concentration of 0.1% (w/w) have a zeta potential between −27.3 and −40.5 mV, so they have a negative charge (anionic hydrocolloids). High absolute values of zeta potential mean show better stability because of the mutual repulsion between theelectrical double layers of macromolecules (Acedo‐Carrillo et al., 2006). The values of zeta potential higher than +30 or lower than −30 indicate a stable solution (McClements, 2015). By increasing the temperature from 40 to 80℃ in HD method, the zeta potential increased from −40.5 to −38.5 mV, so the stability of solution decreased, but no significant difference was observed between them (*p* > .05). The zeta potential in HD samples is lower than −30 mV (−40.5, −39.9, and −38.5 mV), but the zeta potential in foam‐mat drying method is higher than −30 mV (−27.3 mV) with a significant difference, which means that the solutions of HD method are more stable. The conductivity of samples in oven drying method is between 0.172 and 0.183 mS/cm, but in foam‐mat drying method is 0.194 mS/cm with a significant difference. Generally, all the drying methods have good conductivities.

**Table 1:** Zeta potential and conductivity of cress seed gum solutions (0.1%)

Drying method	Zeta (Mv)	Conductivity (mS/cm)
HD 40°C	−40.5 ± 1.2^c^	0.173 ± 0.001^c^
HD 60°C	−39.9 ± 1.0^bc^	0.172 ± 0.002^c^
HD 80°C	−38.5 ± 1.2^b^	0.183 ± 0.001^b^
Foam‐mat D	−27.3 ± 0.1^a^	0.194 ± 0.001^a^

Note

HD (hot air drying).

Different letters indicate significant differences between samples at *p* < .05.

### 3.3 Color measurement

One of the important parameters of quality is color measurement that can be helpful in both processing and raw foods. The color difference between different drying conditions is displayed in Table 2. The a* value for red colors is positive while negative for green colors. The b* value for yellow colors is positive while negative for blue colors. The L* value indicates lightness. The lightness of HD 80 ℃ sample has increased slowly, but the b* value has decreased. The HD 40 ℃ sample has a significant difference with others (*p* < .05). The amount of chroma in HD method is between 0.54 and 0.85, and HD 80℃ has the lowest chroma content. Also, the BI* index for HD 80℃ is lower than 40 and 60℃. CSG powder in HD method has a hue angle between 78.52 and 85.24º which was in the range of red hue to yellow hue (0–90º), but the hue angle of foam‐mat drying method was in the range of yellow to bluish‐green (90–180º). The hue angle is used for food packaging and measuring the light sensitivity of packaging. Total color difference is the difference of stored and control samples (Patras, Brunton, Tiwari, & Butler, 2011). The ΔE* value of CSG powder in oven drying was decreased by increasing temperature. Generally, the foam‐mat drying method has the highest L* value due to shorter drying time but lower values of a*, b*, C*, BI*, and ΔE* compared to oven drying method, so the powder obtained from this method is better.

**Table 2:** Effects of drying methods on the color characteristics of cress seed gum (CSG)

Drying method	L*	a*	b*	BI*	C*	HUE*	ΔE*
HD 40°C	99.68 ± 0.02^c^	0.07 ± 0.01^b^	0.85 ± 0.05^a^	0.88 ± 0.05^a^	0.85 ± 0.05^a^	85.24 ± 0.46^b^	19/00 ± 0.05^a^
HD 60°C	99.72 ± 0.04^bc^	0.10 ± 0.02^a^	0.54 ± 0.06^b^	0.61 ± 0.07^b^	0.55 ± 0.06^b^	79.65 ± 0.71^c^	8.97 ± 0.04^b^
HD 80°C	99.73 ± 0.01^b^	0.11 ± 0.01^a^	0.53 ± 0.02^b^	0.60 ± 0.02^b^	0.54 ± 0.02^b^	78.52 ± 0.70^c^	8.99 ± 0.01^b^
Foam‐mat	99.89 ± 0.01^a^	−0.05 ± 0.007^c^	0.17 ± 0.02^c^	0.13 ± 0.01^c^	0.17 ± 0.02^c^	106.00 ± 2.02^a^	2.99 ± 0.01^c^

Note

HD (hot air drying).

Different letters indicate significant differences between samples at *p* < .05.

### 3.4 Rheological measurements

#### 3.4.1 Shear rate dependency

**Figure 2:**
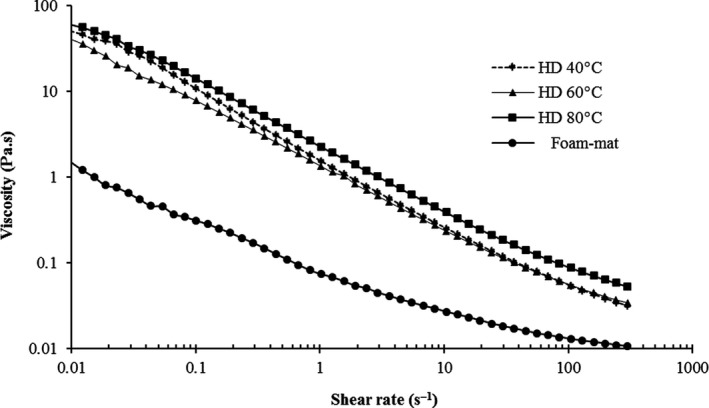
Influence of drying methods on apparent viscosity of cress seed gum. HD (hot air drying)

Different drying conditions such as hot air oven (40, 60 and 80℃) and foam‐mat drying influenced on the rheological characterization of CSG. The flow curves versus shear rate (0.01–300/s) for CSG solution with non‐shear‐thinning behavior are shown in Figure 2. The flow index values of the samples are <1 and are between 0.459 and 0.790, which confirms the pseudoplastic behavior of the cress seed gum. In addition, researchers (Behrouzian, Razavi, & Karazhiyan, 2013; Naji & Razavi, 2014) reported the shear‐thinning behavior of CSG (1%). This shear‐thinning behavior is related to the number of chain entanglements, high molecular weight, and semirigid chain structure (Song, Kim, & Chang, 2006). The fitting of shear stress–shear rate data for CSG solutions is explained in Table 3. The Herschel–Bulkley was the best model among all rheological models due to higher *R* ^2^ value (*R* ^2^ > 0.99). The range of k in power law model was between 0.041 and 1.847, indicating that the viscosity of CSG solution changed significantly with temperature of drying (*p* < .05). High amount of viscosity for HD 80℃ solution in all rheological models exhibited that high drying temperature was more important which is confirmed by high obtained amount for k. These results are consistent with findings of (Naji, Razavi, & Karazhiyan, 2012), which showed a significant increase in consistency coefficient (k) of CSG after thermal treatment. The effect of thermal treatment on the flow behavior of CSG is not clear which is associated with the stability of shear‐thinning behavior. This result is consistent with the results of the Pseudomonas oleovorans (EPS) gum solution by Freitas et al. (2009). For example, the k index in the power law model for heated samples of cress seed gum (1%) is between 0.89 and 1.01, which is lower than the reported value for cress seed gum (1.5%) in the HD method; as a result, viscosity has increased with drying. Also, the flow index in the HD method is lower than the heated samples of cress seed gum, so the HD samples have a higher shear‐thinning behavior (Naji et al., 2012). The viscosity of CSG enhanced with heat treatment due to an irreversible intermolecular arrangement that happened. What is more, this different result may be related to different varieties, plant growth conditions, and extraction process. The molecular weight of polysaccharide changed with different drying conditions and the viscosity of gum solution exaggerated in turn (Nep & Conway, 2011). Khalil and Mohamed Jan (2012) found that the point that the fluid begins to move with the lowest amount of force is called yield stress. The amounts of yield stress (τ_0H_ = 0.033–1.062 Pa), consistency coefficient (*k*_H_ = 0.034–0.914 Pa s^n^), and *n* value (0.459‐0.790) in Herschel–Bulkley model improved when the temperature of drying was enhanced. The rheological parameters in foam‐mat drying showed that the k index, yield stress, and viscosity except *n* value decreased in comparison with HD drying due to the fact that time of drying is the least in foam‐mat drying which causes low degradation. According to the Bingham model, the ranges of τ_0B_ (0.091–2.293 Pa) and *η_B_* (0.011–0.053 pa s) are increased with increase in temperature. The yield stress of HD 80℃ is higher than the others significantly (*p* < .05).

**Table 3:** Rheological parameters of CSG at different drying temperatures

Model	HD 40°C	HD 60°C	HD 80°C	Foam‐mat
Power law model				
K_p_ (Pa s^n^)	1.641 ± 0.258^a^	1.037 ± 0.027^b^	1.847 ± 0.023^a^	0.041 ± 0.003^c^
*n_p_*	0.323 ± 0.031^c^	0.381 ± 0.002^b^	0.357 ± 0.002^b^	0.757 ± 0.009^a^
*R* ^2^	0.976	0.979	0.976	0.999
RMSE	0.372	0.341	0.576	0.027
Herschel–Bulkley model				
*k_H_* (Pa × s^n^)	0.750 ± 0.150^b^	0.550 ± 0.020^c^	0.914 ± 0.019^a^	0.034 ± 0.002^d^
*n_H_*	0.459 ± 0.018^c^	0.491 ± 0.003^b^	0.478 ± 0.004^b^	0.790 ± 0.006^a^
τ_0H_ (Pa)	0.955 ± 0.144^a^	0.584 ± 0.004^b^	1.062 ± 0.003^a^	0.033 ± 0.004^c^
*R* ^2^	0.991	0.991	0.991	0.999
RMSE	0.215	0.222	0.363	0.018
Bingham model				
τ_0B_ (Pa)	1.944 ± 0.356^b^	1.337 ± 0.029^c^	2.293 ± 0.017^a^	0.091 ± 0.007^d^
*η_B_* (pa s)	0.039 ± 0.012^b^	0.034 ± 0.001^b^	0.053 ± 0.000^a^	0.011 ± 0.000^c^
*R* ^2^	0.900	0.907	0.902	0.988
RMSE	0.725	0.712	1.160	0.079
Casson model				
τ_0_ *_c_* (Pa)	3.164 ± 0.748^b^	2.675 ± 0.058^b^	4.587 ± 0.033^a^	0.182 ± 0.014^c^
η_c_ (pa s)	0.015 ± 0.001^b^	0.019 ± 0.000^a^	0.019 ± 0.000^a^	0.018 ± 0.000^a^
*R* ^2^	0.898	0.907	0.902	0.988
RMSE	0.604	0.712	1.160	0.079

Note

HD (hot air drying).

Different letters indicate significant differences between samples at *p* < .05.

#### 3.4.2 Small dynamic oscillatory measurements

##### Strain sweep

**Figure 3:**
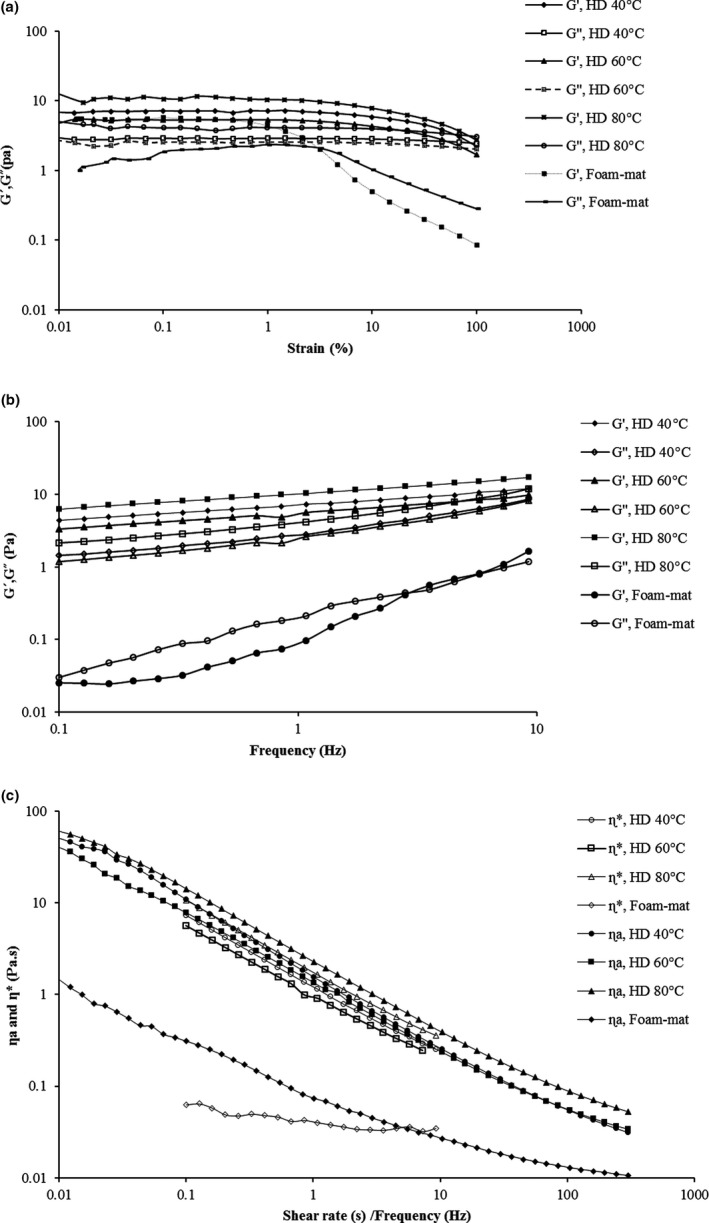
(a) Strain sweep dependency of storage (G′) and loss modulus (G″) at frequency = 1Hz at 20°C, (b) frequency sweep test of G′ and G″, and (c) Cox–Merz curve of cress seed gum (CSG) solution (1.5%). HD (hot air drying)

The area where G´ and G˝ were stable with small deformation is known as linear viscoelastic area. As shown in Figure 3a, when G´ and G˝ begin to reduce, a nonlinear area is formed. The critical strain region (γ_LVE_) is where storage modulus starts to decrease. Therefore, gum deformability is defined as a critical point. The linear region for strong gum solutions was greater in comparison with weak gum solutions (Steffe, 1996). Gel solutions have greater linear area than concentrate solutions, and this is lower than dilute solutions, whereas the natural biopolymer and gel‐sand colloidal gels have LVE > 1 and LVE > 0.1, respectively. The storage ($G_{\text{LVE}}^{\prime }$) and loss modulus (G˝_LVE_) for all solutions in the linear area showed solid‐like behavior. Liquid behavior occurred when G˝_LVE_ and G′_LVE_ crossed over each other (G˝>G′). But the foam‐mat drying displayed liquid behavior in the linear viscoelastic area. The parameters of strain sweep (G_f_, τ_f_, τ_y_, Tan(δ), G˝_LVE_, $G_{\text{LVE}}^{\prime }$, Y_LVE_) are shown in Table 4. As can be seen in Table 4 when the temperature of drying CSG with a concentration of 1.5% is increased, the amount of $G_{\text{LVE}}^{\prime }$is increased too. The strength of the gel samples can be recognized with the amount of $G_{\text{LVE}}^{\prime }$ (Behrouzian et al., 2013). Based on this result, increasing the temperature in HD method developed the gel strength. The $G_{\text{LVE}}^{\prime }$of 80°C (9.68 pa) in HD method is higher than 40°C and 60°C. Also, the amount of G˝_LVE_ in the linear region increased when the temperature in HD method was enhanced. Critical strain (Y_L_) for foam‐mat drying method (1.48%) was lower than HD method, which showed that this sample had a lower resistance below strain amplitude (*p* < .05). (Tan(δ) = G˝/G′) is for assessment of physical properties of the samples. A Tan(δ) < 1 represents an elastic behavior, but a Tan(δ) > 1 represents a viscous behavior. Tan(δ) values higher than 0.1 define that the samples are not true gel, and their structure is between concentrated biopolymer and real gel. The amounts of Tan(δ) for different drying conditions were between (0.43 and 0.63) where amounts lower than 1 and higher than 0.1 show the existence of elastic structure in weak biopolymer gel (Table 4). Dried samples with different methods were not real gels and they can be reflected as “weak gels” which were formed by entangled flexible random‐coil chains (Morris, 1990; Naji‐Tabasi et al., 2016). The point that G′ and G″ are equal (G_f_) is a suitable indicator of structural strength, and the HD 80℃ method had the highest strength (3.21 pa). The standard of structural strength and the shape preservation factor versus mechanical stresses are determination of the critical strain in the linear viscoelastic range. Nonlinear variations in the structure are called yield stress which corresponds to this strain. The flow‐point stress (τ_f_) and yield stress (τ_y_) were higher for HD 80℃ method. Yield stress can be beneficial when gums are used as a binder to keep food formulations in place. The rheological techniques and assumptions used in assessment are applied to introduce the yield stress, and the amount of yield stress depends on the evaluation techniques (Steffe, 1996).

**Table 4:** Storage modulus ($G_{\text{LVE}}^{\prime }$), loss modulus, the critical strain (Υ_L_%), loss tangent (tanδ_LVE_), flow‐point stress (τ*_f_*), and yield stress (τ_y_) in the linear viscoelastic region, and corresponding modulus (G_f_: G′ = G″) in the strain sweep test at frequency (1 HZ), frequency sweep parameters (*G*′, G″, complex viscosity (ɳ*), Tan(δ), and slope of ɳ*) at frequency (1Hz), strain (0.3%), and frequency dependency of the storage modulus (*G*′) and loss (*G*″) for CSG 1.5% at 20℃

Parameter	HD 40°C	HD 60°C	HD 80°C	Foam‐mat
Strain sweep parameters				
Y_L_ (%)	3.15 ± 0.01^a^	3.15 ± 0.01^a^	3.13 ± 0.01^a^	1.48 ± 0.52^b^
Tan(δ)	0.43 ± 0.01^a^	0.50 ± 0.01^a^	0.43 ± 0.00^a^	0.63 ± 0.37^a^
$G_{\text{LVE}}^{\prime }$ (pa)	6.79 ± 0.45^b^	5.11 ± 0.04^c^	9.68 ± 0.84^a^	3.67 ± 0.17^d^
*G*_f_ (pa)	2.49 ± 0.08^ab^	2.11 ± 0.03^b^	3.21 ± 0.10^a^	2.12 ± 0.88^b^
$G_{\text{LVE}}^{\prime \prime }$ (pa)	2.90 ± 0.13^b^	2.54 ± 0.02^bc^	4.14 ± 0.36^a^	2.32 ± 0.32^c^
τ*_y_* (pa)	0.23 ± 0.01^b^	0.18 ± 0.00^c^	0.33 ± 0.03^a^	0.06 ± 0.04^d^
τ*_f_* (pa)	3.02 ± 0.13^b^	2.37 ± 0.02^c^	3.76 ± 0.10^a^	0.08 ± 0.01^d^
Frequency dependency of G′				
a	7.08 ± 0.09^b^	5.53 ± 0.13^c^	10.23 ± 0.08^a^	0.11 ± 0.02^d^
b	0.22 ± 0.06^b^	0.24 ± 0.04^b^	0.22 ± 0.05^b^	1.21 ± 0.16^a^
*R* ^2^	0.97	0.99	0.99	0.99
RMSE	0.28	0.17	0.32	0.04
Frequency dependency of G″				
c	2.92 ± 0.00^b^	2.81 ± 0.11^b^	4.22 ± 0.09^a^	0.20 ± 0.01^c^
d	0.44 ± 0.00^b^	0.46 ± 0.01^b^	0.43 ± 0.00^b^	0.80 ± 0.05^a^
*R* ^2^	0.99	0.99	0.98	1.00
RMSE	0.23	0.23	0.33	0.02
Frequency sweep parameters				
*G*′ (pa)	7.28 ± 0.19^a^	5.60 ± 0.20^c^	10.35 ± 0.25^a^	0.10 ± 0.09^d^
*G*″ (pa)	2.78 ± 0.11^b^	2.61 ± 0.10^c^	4.15 ± 0.09^a^	0.21 ± 0.03^d^
Tan(δ)	0.38 ± 0.02^b^	0.47 ± 0.00^b^	0.40 ± 0.00^b^	2.17 ± 1.04^a^
ɳ* (Pa s)	1.15 ± 0.03^b^	0.91 ± 0.03^c^	1.64 ± 0.04^a^	0.04 ± 0.03^d^
Slope of ɳ*	**−**0.79 ± 0.02^b^	**−**0.82 ± 0.05^b^	**−**0.78 ± 0.01^b^	**−**0.01 ±0.01^a^

Note

HD (hot air drying).

Different letters indicate significant differences between samples at *p* < .05.

##### Frequency sweep

Selecting of solutions based on frequency sweep is as follows: strong and weak gels, dilute solutions, and entanglement network system. Figure 3b shows that the G′ and G″ have low dependence on the frequency and G′ is higher than G″; as a result, the samples have weak gel‐like behavior. At low frequencies, G′ is larger than G″, and two modules are divergent, but with increasing frequency, they get closer together. These results were in compliance with the findings of Karazhiyan et al. (2011). The factors that the frequency sweep depends on are polymer concentration, molecular weight, dispersity, structural characteristics, and solvent quality. The power law equation is used to explain the dependence on the frequency of G′ and G″.

(16) $$G^{\prime }=a.\;\omega^{b}$$

(17) $$G^{\prime \prime }=c.\;\omega^{b}$$

where a and c are constants, b and d are related to frequency exponents and give information about the viscoelastic nature of food, and ω is the angular frequency (Özkan, Xin, & Chen, 2002). The power law parameters are summarized in Table 4. The slopes were b from 0.22 to 1.21 and d from 0.43 to 0.80 which were both positive, so the samples had gel‐like behavior. b represents the strength and nature of the gel where if b = 0, the gel is covalent, if b > 0, indicates the physical gel, if close to zero, the storage modulus cannot change with the frequency, and when close to 1, the gel is viscous. As the frequency increases, the loss modulus changes more than the storage modulus, due to the fact that d is greater than b. The foam‐mat drying method has the highest b and d values, so it has higher frequency sensitivity than other drying methods. The value of b is 1.21, which indicates that the reaction of the polysaccharide chain is weak. By increasing drying temperature, the amount of a has increased, so the HD 80°C has the highest a (10.23); as a result, the power of the gel network becomes stronger. Also, it has a structure with more elasticity than the others. The samples exhibited weak gel behavior, which higher value of a than c proves it (Razavi, Cui, & Ding, 2016). In Figure 3b, in oven‐dried samples, the storage modulus is higher than loss modulus, and samples have a solid behavior, while in foam‐mat drying process, the storage modulus is lower than the loss modulus. The loss tangent is used to study the viscoelastic feature, which depends on the lost energy in each cycle divided by the stored energy in each cycle. For dilute solutions, the amount of Tan(δ) is very high, for amorphous polymers between 0.2 and 0.3, and for glass crystal polymers (close to 0.01). If Tan(δ) is less than 1, it indicates elastic behavior, and if greater than 1, it represents viscous behavior (Steffe, 1996). In hot air drying method, the Tan(δ) value was between 0.38 and 0.47, indicating that the samples have more elastic behavior. Drying has increased the amount of elastic modulus (G′) and viscous modulus (G″), and the HD 80°C method has the largest G´ and G˝. CSG solutions have non‐Newtonian shear‐thinning behavior, because the complex viscosity decreases with increasing frequency. Drying affects the complex viscosity and increases as temperature rises from 40 to 80°C. A polysaccharide with weak gel properties has a complex viscosity slope of about −0.76, which is formed by overlapping and random chain interactions (Morris, 1990). A good elastic system has a slope of −1 and for an elastic system, the G* is nondependent on ω (ɳ* = G*/ω). The Log ɳ* of the complex viscosity with a greater slope in front of the ω indicates that the gel is more elastic. The samples have a slope of more than −0.76, and there is no significant difference between them.

#### 3.4.3 Relationship between steady and dynamic shear rheology

Comparison the changes of the complex viscosity variations as a function of angular frequency with the apparent viscosity as a function of shear viscosity is called Cox–Merz rule. The relationship between shear and dynamic viscosity at a shear rate (0.01–300/s) and a frequency (0.1–10) Hz was described using the Cox–Merz rule. This rule applies to random‐coil biopolymer solutions. A greater complex viscosity than apparent viscosity indicates weakness of the gel, but in Figure 3c, apparent viscosity is higher than complex viscosity. This may be related to different types of molecular rearrangement (Richardson & Ross‐Murphy, 1987). Thus, the graph shows a strong network that remains intact in the continuous shear, but has been damaged in an amplitude oscillatory (Fang, Takemasa, Katsuta, & Nishinari, 2004).

### 3.5 Textural characteristics

**Figure 4:**
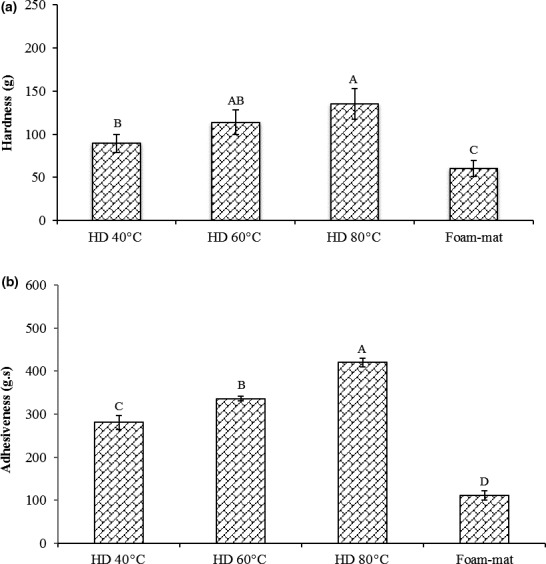
Influence of drying methods on textural properties of cress seed gum gel. (a) hardness; (b) adhesiveness. HD (hot air drying). Columns with different letters are significantly different (*p* < .05)

The effect of different drying methods on the hardness of cress seed gel is shown in Figure 4a. Hardness is the force needed to get deformation and demonstrates the gel strength. The temperature has positive effects on the hardness of cress seed gel. There is no significant difference between values of hardness of cress seed gel at 40, 60, and 80°C in HD method. The HD 80℃ has the highest amount of hardness. One of the most important roles that junction zones play a very important role in the gelling process of hydrocolloids. The junction zones affect the thermal behavior of gels. The length of the junction zones is one of the most important factors that affect their strength. An important determinant of gel property is the number of molecules that form junction zones. As the number of molecules in the junction zones increases, the gel will be more rigid (Saha & Bhattacharya, 2010). Roopa and Bhattacharya (2010) reported the effect of different heat treatment on the strength of alginate gel and concluded that hardness of alginate gel increases by heating.

Adhesiveness can be beneficial for CSG, when soluble in water, because it facilitates binding and agglomerates powders. The work required for separating the surface of the food is called adhesiveness. With increasing the temperature in oven drying method, the adhesiveness of cress seed gel is significantly increased. Adhesiveness can have positive and negative effects (Meiron & Saguy, 2007). The adhesiveness can be recognized with adhesion to manufacturing tools and sticking to fingers. Adhesion of products in some cases such as salad dressing, puddings, confectionary, and bakery has positive effects, but for spaghetti and other pasta products, bread crumbs and some meat products has negative effects. In this research, the cress seed gel has a high value of adhesiveness in different drying conditions, so this gum can be useful in salad dressing. Most consumers prefer a salad dressing which does not precipitate.

### 3.6 Emulsifying properties

The preparation of emulsion is one important character of hydrocolloids, whereas just some of them can perform as emulsifying agents (gum Arabic, hydrophobic modified starches, modified celluloses, some kinds of pectin, and some galactomannans like guar, LBG, fenugreek). Good capacity of emulsion was observed in all samples (>90%). This behavior was recognized as a result of existence of protein factor. As a result, the viscosity of aqueous phase increased and affected the rheological variation of the continuous phase thus delaying the droplets movement (Naji et al., 2012). In HD method, the emulsion capacity of CSG decreased with increasing temperature, although no significant changes were observed. This research outcome was in agreement with Garti, Slavin, and Aserin (1999) who described that thermal treatment decreases the emulsion capacity of gum Arabic solution due to proteins denaturated by heating the gum. By heating at 80°C for 30 min, all emulsions were stable, and no significant difference was observed between them. CSG has more emulsion stability than Lepidium perfoliatum seed gum, Lallemantia royleana seed gum, and salvia macrosiphon seed gum, guar, xanthan, and locust bean gum (Razavi, 2019).

### 3.7 Foaming properties

**Figure 5:**
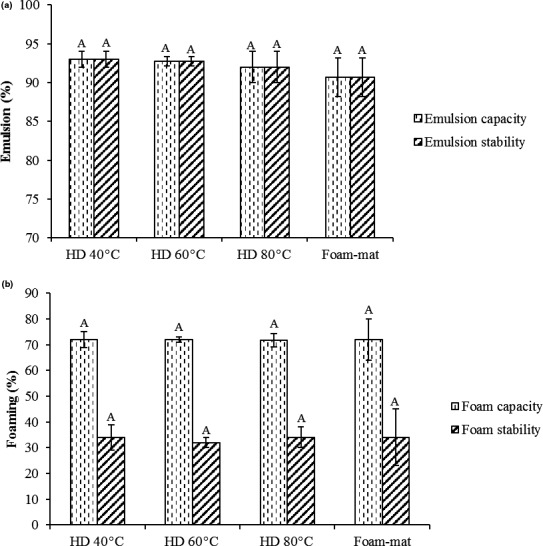
Effects of drying methods on emulsifying and foaming properties of cress seed gum (CSG). (a) emulsion capacity and emulsion stability; (b) foaming capacity and foaming stability). HD (hot air drying). Columns with different letters are significantly different (*p* < .05)

Dispersed gas in the liquid in high volume is referred to foam, which is used in various products including beer, whipped cream, ice cream, meringue, and marshmallow (Indrawati, Wang, Narsimhan, & Gonzalez, 2008). An important group of food foams are components derived from egg proteins which form liquid–gas systems. In some cases, mono‐, di‐, and polysaccharides additives are used to prevent foam decomposition. The initial volume (capacity of the foam) and the volume lost over time (foam stability) are known as foam properties. The capacity and stability of foam are specified in Figure 5b. The amount of foam capacity of CSG in oven and foam‐mat drying methods is (>70%), which indicates that CSG has a good foaming capacity. But after 30 min, the volume of the foam has dropped to 30%. CSG can reduce surface tension and has high foam ability, since it is present in the glycoprotein complex and shows a high foaming capacity. Drying has reduced the foaming capacity, so the HD 80°C has a lower foaming capacity than 40 and 60°C, but there is no significant difference between them.

## 4 CONCLUSION

The results of this study indicate that pseudoplastic behavior of CSG solution has been considered in steady and dynamic shear evaluation. The Herschel–Bulkley's model is the best model. As the temperature was increased in HD method, the viscosity was increased too. Increasing in temperature did not have a destructive effect on CSG, indicating the irreversible structure of the gum. Also, dynamic tests show that this gum has a weak gel behavior. HD samples have more elastic structure and strength than the foam‐mat method because they have higher storage modulus values, especially at 80°C. The temperature has a significant effect on texture properties where hardness and adhesion were increased with increasing the temperature of drying. The FT‐IR test confirmed the nature of polysaccharide gum. The characteristics of emulsion, foaming, and zeta potential were not significantly changed. Overall, the HD method has better rheology and textural properties in comparison with foam‐mat drying method. Therefore, CSG can be selected in high‐temperature industrial processes such as pasteurization, cooking, and sterilization. It can also be used as a suitable alternative for plant hydrocolloids in food products.

## CONFLICT OF INTEREST

The authors have declared no conflict of interest.

## ETHICAL APPROVAL

This study does not involve any human or animal testing.
